# Tacrolimus trough levels higher than 6 ng/mL might not be required after a year in stable kidney transplant recipients

**DOI:** 10.1371/journal.pone.0235418

**Published:** 2020-07-02

**Authors:** Hee-Yeon Jung, Min Young Seo, Yena Jeon, Kyu Ha Huh, Jae Berm Park, Cheol Woong Jung, Sik Lee, Seung-Yeup Han, Han Ro, Jaeseok Yang, Curie Ahn, Ji-Young Choi, Jang-Hee Cho, Sun-Hee Park, Yong-Lim Kim, Chan-Duck Kim

**Affiliations:** 1 Department of Internal Medicine, School of Medicine, Kyungpook National University, Kyungpook National University Hospital, Daegu, South Korea; 2 Department of Internal Medicine, Pohang St. Mary’s Hospital, Pohang, South Korea; 3 Department of Statistics, Kyungpook National University, Daegu, South Korea; 4 Department of Surgery, Yonsei University College of Medicine, Seoul, South Korea; 5 Department of Surgery, Sungkyunkwan University, Seoul Samsung Medical Center, Seoul, South Korea; 6 Department of Surgery, Korea University College of Medicine, Seoul, South Korea; 7 Department of Internal Medicine, Chonbuk National University Hospital, Jeonju, South Korea; 8 Department of Internal Medicine, Keimyung University, Dongsan Medical Center, Daegu, South Korea; 9 Department of Internal Medicine, Gachon University, Gil Hospital, Incheon, South Korea; 10 Department of Surgery, Seoul National University College of Medicine, Seoul, South Korea; 11 Department of Internal Medicine, Seoul National University College of Medicine, Seoul, South Korea; International University of Health and Welfare, School of Medicine, JAPAN

## Abstract

**Background:**

Little is known regarding optimal tacrolimus (TAC) trough levels after 1 year post-transplant in stable kidney transplant recipients (KTRs) who have not experienced renal or cardiovascular outcomes. This study aimed to investigate the effect of 1-year post-transplant TAC trough levels on long-term renal and cardiovascular outcomes and opportunistic infections in stable KTRs.

**Methods:**

KTRs receiving TAC with mycophenolate-based immunosuppression who did not experience renal or cardiovascular outcomes within 1 year post-transplant were enrolled from a multicenter observational cohort study. Renal outcome was defined as a composite of biopsy-proven acute rejection, interstitial fibrosis and tubular atrophy, and death-censored graft loss. Cardiovascular outcome was defined as a composite of de novo cardiomegaly, left ventricular hypertrophy, and cardiovascular events. Opportunistic infections were defined as the occurrence of BK virus or cytomegalovirus infections.

**Results:**

A total of 603 eligible KTRs were divided into the low-level TAC (LL-TAC) and high-level TAC (HL-TAC) groups based on a median TAC level of 5.9 ng/mL (range 1.3–14.3) at 1 year post-transplant. The HL-TAC group had significantly higher TAC trough levels at 2, 3, 4, and 5 years compared with the levels of the LL-TAC group. During the mean follow-up of 63.7 ± 13.0 months, there were 121 renal outcomes and 224 cardiovascular outcomes. In multivariate Cox regression analysis, LL-TAC and HL-TAC were not independent risk factors for renal and cardiovascular outcomes, respectively. No significant differences in the development of opportunistic infections and de novo donor-specific anti-human leukocyte antigen antibodies and renal allograft function were observed between the two groups.

**Conclusions:**

TAC trough levels after 1 year post-transplant remained at a similar level until the fifth year after kidney transplantation and were not directly associated with long-term outcomes in stable Korean KTRs who did not experience renal or cardiovascular outcomes. Therefore, in Asian KTRs with a stable clinical course, TAC trough levels higher than approximately 6 ng/mL might not be required after a year of kidney transplantation.

## Introduction

Underdosing of tacrolimus (TAC) in kidney transplant recipients (KTRs) can lead to biopsy-proven acute rejection (BPAR) and immunologic sensitization; however, overdosing of TAC can result in calcineurin inhibitor (CNI) toxicity and opportunistic infections including BK virus and cytomegalovirus (CMV) infections, which have detrimental effects on renal allograft outcomes [1–5]. Furthermore, CNI exposure can increase the risk of new-onset diabetes mellitus, hypertension, and lipid dysregulation, which are considered as potential risk factors for cardiovascular disease [6]. Therefore, the maintenance of optimal TAC trough levels is crucial to improve transplant outcomes.

Optimal TAC trough levels may be different according to the post-transplant period. Previous studies have reported an association between TAC trough levels within 1 year post-transplant and kidney transplantation outcomes [7–20]. The Kidney Disease: Improving Global Outcomes guidelines suggest that 5–15 ng/mL of TAC trough levels should be maintained during the first 2–4 months post-transplant and then reduced in stable KTRs to minimize toxicity, with a low quality of evidence [21]. However, little is known regarding optimal TAC trough levels after 1 year post-transplant in stable KTRs who have not experienced renal or cardiovascular outcomes. Furthermore, since ethnicity can affect tacrolimus pharmacokinetics [22], it is crucial to determine the optimal TAC trough levels in Asian KTRs.

This study aimed to investigate the effect of 1-year post-transplant TAC trough levels on renal and cardiovascular outcomes in stable Korean KTRs who did not experience renal or cardiovascular outcomes within 1 year post-transplant.

## Materials and methods

### Participants

*De novo* KTRs were enrolled from the Korean Cohort Study for Outcome in Patients with Kidney Transplantation (KNOW-KT) between 2012 and 2016 and followed up until 2019. Out of 1,080 KTRs, we included 707 KTRs receiving TAC with mycophenolate-based immunosuppression at 1 year. Overall, 101 KTRs who experienced renal or cardiovascular outcomes within 1 year post-transplant (renal = 94, cardiovascular = 33, both = 26), 1 patient with TAC trough levels > 20 ng/mL, and 2 patients with insufficient information were excluded. As a result, 603 KTRs were included in this study. The Institutional Review Committee of each participating center approved the KNOW-KT study protocol [Chonbuk National University Hospital; Gachon University Gil Medical Center; Keimyung University Dongsan Hospital; Korea University Anam Hospital; Kyungpook National University Hospital; Samsung Medical Center, Seoul; Seoul National University Hospital; Yonsei University, Severance Hospital (in alphabetical order)] [23]. All patients provided their written informed consent before participating in the study. All clinical investigations were conducted in accordance with the guidelines of the 2008 Declaration of Helsinki.

### Variables

TAC trough levels and TAC dosages were recorded at 1 year and annually thereafter. TAC trough levels and TAC dosages were determined by the physicians’ clinical judgment. Eligible KTRs were divided into the low-level TAC (LL-TAC) and high-level TAC (HL-TAC) groups based on a median TAC level of 5.9 ng/mL (range 1.3–14.3) at 1 year post-transplant.

Possible confounders for renal composite endpoints included TAC trough level, TAC dosage, age, sex, body mass index (BMI), number of human leukocyte antigen (HLA) mismatches, type of transplant donor, re-transplantation, and desensitization. Possible confounders for cardiovascular composite endpoints included TAC trough levels, TAC dosage, age, sex, BMI, primary kidney disease, dialysis duration, smoking, total cholesterol, high-density lipoprotein (HDL), and systolic blood pressure (BP).

Information on de novo donor-specific anti-HLA antibodies (DSAs) was obtained at baseline, 1 year, and 5 years post-transplant and when clinically indicated.

### Outcomes

Renal outcome was defined as a composite of BPAR, interstitial fibrosis and tubular atrophy (IFTA), and death-censored graft loss after 1 year post-transplant. IFTA grade I, II, and III were included. Cardiovascular outcome was defined as a composite of de novo cardiomegaly on chest X-ray, left ventricular hypertrophy on electrocardiogram, and cardiovascular events after 1 year post-transplant. Chest X-ray and electrocardiogram were performed at baseline and annual visits. Cardiomegaly on chest X-ray was defined as cardiothoracic ratio > 50%. Cardiovascular events were defined as myocardial infarction, unstable angina, percutaneous coronary intervention, coronary artery bypass grafting, or stroke. The development of de novo DSAs and estimated glomerular filtration rates (eGFRs) were compared between the two groups.

Opportunistic infections were defined as the occurrence of BK virus or CMV infections. BK virus infection was defined as an occurrence of BK viremia (≥ 10^4^ copies/mL) or diagnosed biopsy-proven BK virus nephropathy. CMV infection was defined as a presence of significant positive CMV polymerase chain reaction or diagnosed CMV disease.

### Statistical analysis

Continuous variables are expressed as the mean ± standard deviation for normally distributed data and as the median with range when the values were not normally distributed. Differences between the groups were assessed by independent sample *t*-tests and chi-squared tests as appropriate. The Cox regression model was used to analyze the factors associated with the development of renal and cardiovascular outcomes and opportunistic infections. The cumulative incidences of renal and cardiovascular outcomes were analyzed according to TAC trough levels using the Kaplan-Meier method. Statistical analysis was performed using the SAS system for Windows, version 9.2 (SAS Institute Inc., Cary, NC). P values < 0.05 were considered statistically significant.

## Results

### Baseline characteristics

Table 1 shows the baseline characteristics according to a median TAC level of 5.9 ng/mL at 1 year post-transplant. The mean TAC trough levels were 7.8 ± 1.6 and 4.6 ± 1.0 ng/mL in KTRs with HL-TAC and LL-TAC, respectively. In comparison with patients with HL-TAC, those with LL-TAC were significantly younger (46.6 ± 11.2 vs. 44.7 ± 11.4 years, P = 0.041) and received less anti-thymocyte globulin as induction therapy (14.7 vs. 6.6%, P = 0.001). There were no significant differences in TAC dose, mycophenolic acid dose, the use of steroid, recipient sex, donor age, donor sex, BMI, primary kidney disease, dialysis duration, type of transplant donor, re-transplantation, number of HLA mismatches, panel reactive antibody positivity, desensitization, smoking, lipid profile, BP, the use of angiotensin-converting enzyme inhibitors, angiotensin II receptor blockers, and statins, DSAs at 1 year, and eGFR at 1 year.

**Table 1:** Baseline characteristics.

Variables	TAC > 5.9 ng/mL (n = 299)	TAC ≤ 5.9 ng/mL (n = 304)	P value
TAC trough levels at 1 year, ng/mL	7.8 ± 1.6	4.6 ± 1.0	< 0.001
TAC dose at 1 year, mg	3.9 ± 2.5	3.5 ± 1.9	0.065
MMF dose at 1 year, mg	942.5 ± 284.4 (n = 181)	935.4 ± 318.4 (n = 204)	0.819
EC-MPS dose at 1 year, mg	659.5 ± 247.2 (n = 118)	677.8 ± 220.0 (n = 100)	0.567
Use of steroid at 1 year, n (%)			
Yes	270 (90.3)	273 (89.8)	0.838
Missing	29 (9.7)	31 (10.2)	
Recipient age, years	46.6 ± 11.2	44.7 ± 11.4	0.041
Recipient sex, male, n (%)	194 (64.9)	184 (60.5)	0.269
Donor age, years	44.1 ± 11.9	43.8 ± 12.1	0.748
Donor sex, male, n (%)	159 (53.2)	151 (49. 7)	0.389
Recipient BMI, kg/m^2^	22.8 ± 3.5	22.7 ± 3.5	0.512
Primary kidney disease, n (%)			
Diabetes	65 (21.7)	65 (21.4)	0.819
Non-diabetes	196 (65.6)	195 (64.1)	
Unknown	38 (12.7)	44 (14.5)	
Dialysis duration, months	25.1 ± 40.8	27.4 ± 46.3	0.523
Type of donor, n (%)			
Living	246 (82.3)	241 (79.3)	0.350
Deceased	53 (17.7)	63 (20.7)	
Re-transplantation, n (%)	21 (7.0)	19 (6.3)	0.703
Number of HLA mismatches			
Total	3.4 ± 1.6	3.3 ± 1.6	0.706
DR	1.1 ± 0.7	1.1 ± 0.7	0.513
PRA positivity (> 0%), n (%)	93 (31.1)	111 (36.6)	0.152
Desensitization, n (%)	85 (28.6)	66 (21.8)	0.054
Induction therapy, n (%)			
IL-2RB	255 (85.3)	284 (93.4)	0.001
ATG	44 (14.7)	20 (6.6)	
Smoking at KT			
Never	147 (49.2)	168 (55.3)	0.295
Current or former	150 (50.2)	134 (44.1)	
Missing	2 (0.7)	2 (0.7)	
Total cholesterol at 1 year, mg/dL	177.1 ± 34.68	177.7 ± 34.7	0.818
LDL at 1 year, mg/dL	98.2 ± 28.0	98.5 ± 28.6	0.922
HDL at 1 year, mg/dL	56.3 ± 17.1	59.1 ± 17.7	0.052
SBP at 1 year, mmHg	124.7 ± 11.9	123.5 ± 13.0	0.244
DBP at 1 year, mmHg	78.4 ± 10.4	77.7 ± 10.5	0.425
Use of drugs at 1 year, n (%)			
ACE inhibitors/ARBs	42 (14.1)	34 (11.2)	0.300
Statins	132 (44.2)	141 (46.5)	0.556
DSAs at 1 year, n (%)	13 (4.3)	17 (5.6)	0.067

Values are shown as the mean ± standard deviation (range) or number (%).

Abbreviations: ACE, angiotensin-converting enzyme; ARBs, angiotensin II receptor blockers; ATG; anti-thymocyte globulin; BMI, body mass index; DBP, diastolic blood pressure; DSA, donor-specific anti-human leukocyte antigen antibodies; EC-MPS, enteric-coated mycophenolate sodium; HDL, high-density lipoprotein; HLA, human leukocyte antigen; IL-2RB, interleukin-2 receptor blocker; KT, kidney transplantation; LDL, low-density lipoprotein; MMF, mycophenolate mofetil; PRA, panel reactive antibody; SBP, systolic blood pressure; TAC, tacrolimus.

### TAC trough levels and renal outcomes

**Fig 1:**
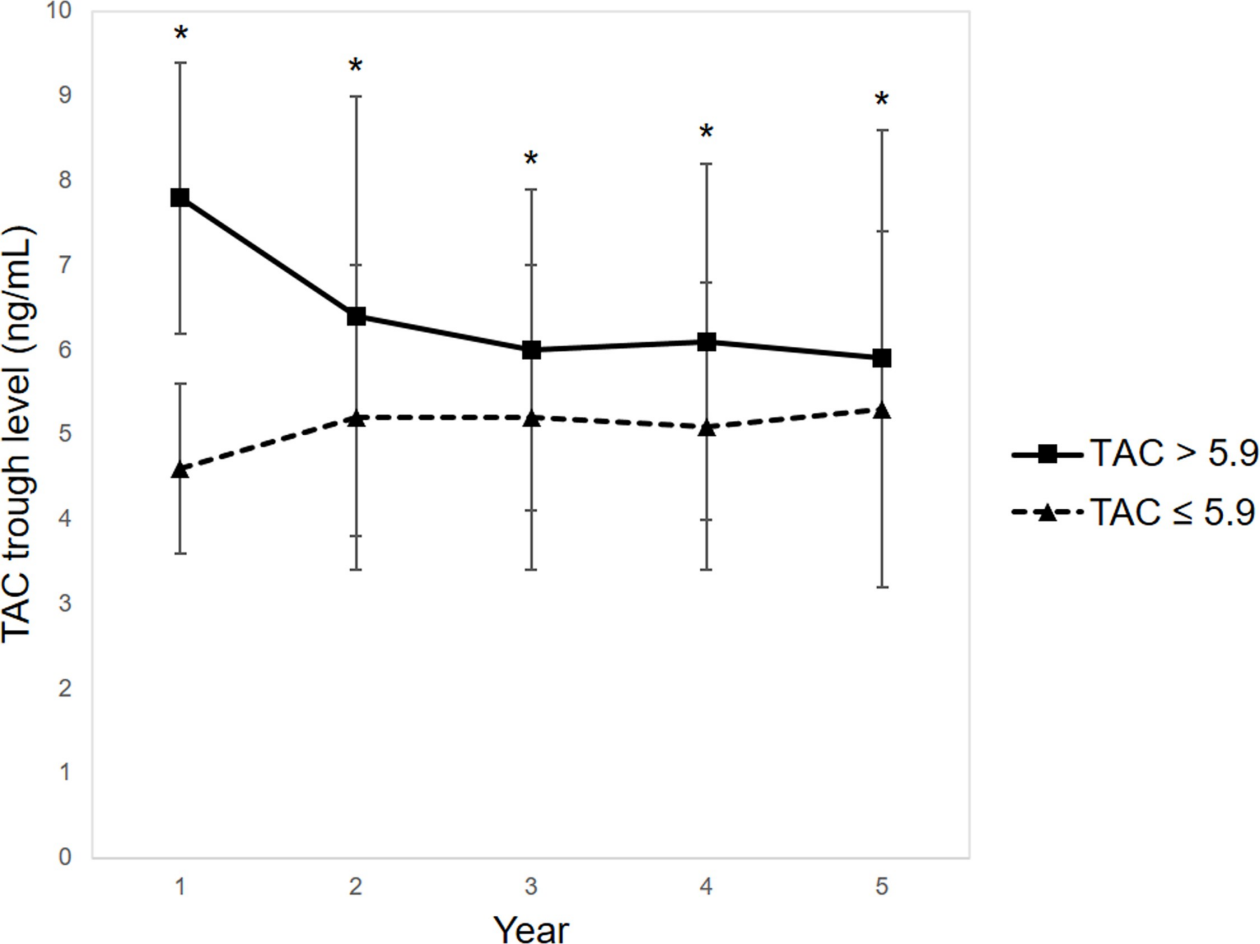
TAC trough levels at 2, 3, 4, and 5 years post-transplant. Patients with HL-TAC at 1 year had significantly higher TAC trough levels at 2 years (6.4 ± 5.2 vs. 5.2 ± 1.8 ng/mL, P < 0.001), 3 years (6.0 ± 1.9 vs. 5.2 ± 1.8 ng/mL, P < 0.001), 4 years (6.1 ± 2.1 vs. 5.1 ± 1.7 ng/mL, P < 0.001), and 5 years (5.9 ± 5.3 vs. 5.3 ± 2.1 ng/mL, P < 0.001) compared with the levels of patients with LL-TAC. *P < 0.001. Abbreviations: LL, low-level; HL, high-level; TAC, tacrolimus.

Patients with HL-TAC at 1 year had significantly higher TAC trough levels at 2 years (6.4 ± 5.2 vs. 5.2 ± 1.8 ng/mL, P < 0.001), 3 years (6.0 ± 1.9 vs. 5.2 ± 1.8 ng/mL, P < 0.001), 4 years (6.1 ± 2.1 vs. 5.1 ± 1.7 ng/mL, P < 0.001), and 5 years (5.9 ± 5.3 vs. 5.3 ± 2.1 ng/mL, P < 0.001) compared with the levels of patients with LL-TAC (Fig 1).

During the mean follow-up of 63.7 ± 13.0 months, 121 renal outcomes occurred after 1 year post-transplant. Detailed information on renal outcomes is presented in S1 Table. Univariate and multivariate Cox regression analysis demonstrated that TAC trough levels, TAC dose, age, sex, BMI, number of HLA mismatches, re-transplantation, and desensitization were not significantly associated with renal composite endpoints (Table 2).

**Table 2:** Univariate and multivariate Cox regression analysis for renal composite endpoints.

	Univariate		Multivariate	
	HR (95% CI)	P value	HR (95% CI)	P value
TAC ≤ 5.9 vs. TAC > 5.9, ng/mL	0.83 (0.58–1.18)	0.298	0.85 (0.59–1.23)	0.389
TAC dose, mg	1.06 (0.99–1.13)	0.079	1.06 (0.99–1.13)	0.079
Age, years	1.00 (0.98–1.01)	0.525	0.99 (0.97–1.01)	0.373
Men vs. women	1.28 (0.87–1.87)	0.207	1.25 (0.84–1.85)	0.272
BMI, kg/m^2^	1.02 (0.97–1.07)	0.521	1.02 (0.97–1.08)	0.489
Number of HLA mismatches				
Total	1.04 (0.93–1.17)	0.475	1.10 (0.90–1.33)	0.354
DR	1.02 (0.79–1.32)	0.889	0.87 (0.56–1.36)	0.534
Deceased vs. living donor	1.19 (0.77–1.82)	0.437	1.27 (0.80–2.01)	0.311
Re-transplantation	1.01 (0.49–2.06)	0.988	0.97 (0.47–2.02)	0.938
Desensitization	1.11 (0.74–1.67)	0.608	1.21 (0.79–1.86)	0.391

Abbreviations: BMI, body mass index; CI, confidence interval; HLA, human leukocyte antigen; HR, hazards ratio; TAC, tacrolimus.

Multivariate Cox regression analysis for BPRA or IFTA are shown in S2 Table. TAC trough levels were not significantly associated with both BPRA and IFTA.

**Fig 2:**
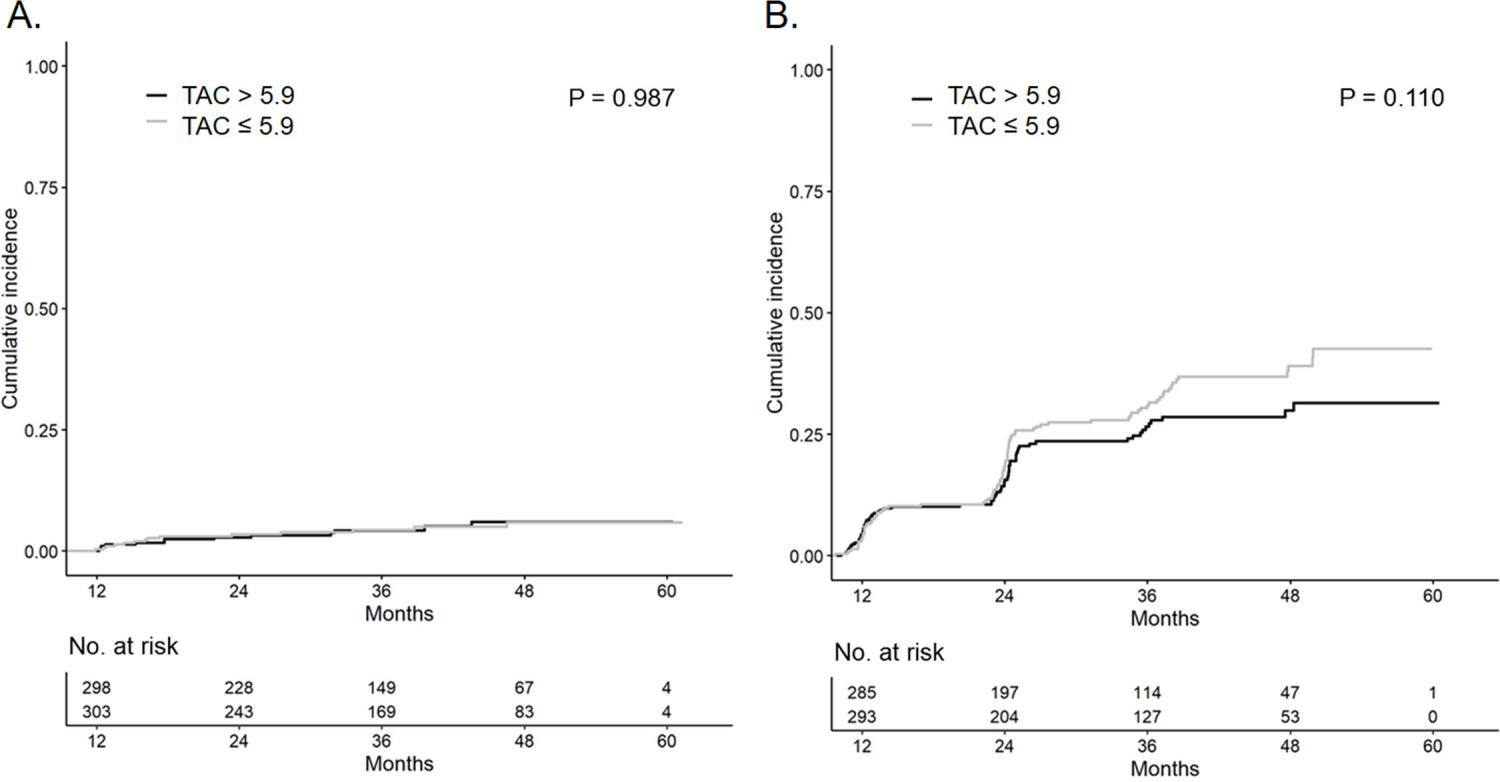
Cumulative incidence of (A) renal composite endpoints and (B) cardiovascular composite endpoints according to TAC trough levels. No significant differences in the development of renal and cardiovascular outcomes between the HL-TAC and LL-TAC groups were observed in the Kaplan-Meier plot. Abbreviations: LL, low-level; HL, high-level; TAC, tacrolimus.

Kaplan-Meier analysis showed that there was no significant difference in the development of renal outcomes between the HL-TAC and LL-TAC groups (Fig 2A).

TAC trough levels of 5.0–5.9, ≤ 5, and ≤ 4 ng/mL compared with > 5.9 ng/mL were not associated with renal composite endpoints even after adjusting for TAC dose, age, sex, BMI, number of HLA mismatches, type of transplant donor, re-transplantation, and desensitization (Table 3). Subgroup analysis including deceased donor and deceased donor with donor age ≥ 60 revealed that renal outcome was not associated with LL-TAC compared with HL-TAC.

**Table 3:** Univariate and multivariate Cox regression analysis for renal composite endpoints in subgroups.

Subdivided TAC trough levels		HR (95% CI)	P value
5.0 < TAC ≤ 5.9 vs. TAC > 5.9, ng/mL	Univariate	1.09 (0.67–1.76)	0.734
	Multivariate*	1.13 (0.69–1.85)	0.617
TAC ≤ 5 vs. TAC > 5.9, ng/mL	Univariate	0.78 (0.51–1.18)	0.231
	Multivariate*	0.74 (0.49–1.13)	0.166
TAC ≤ 4 vs. TAC > 5.9, ng/mL	Univariate	1.01 (0.60–1.70)	0.970
	Multivariate*	1.01 (0.59–1.71)	0.986
Subgroups: TAC ≤ 5.9 vs. TAC > 5.9, ng/mL			
Deceased donor	Univariate	0.75 (0.35–1.60)	0.458
Deceased donor & donor age ≥ 60 years	Univariate	0.62 (0.10–3.75)	0.606

Abbreviations: CI, confidence interval; HR, hazards ratio; TAC, tacrolimus.

*Adjusted for TAC dose, age, sex, BMI, number of HLA mismatches, type of transplant donor, re-transplantation, desensitization, and induction therapy.

### TAC trough levels and cardiovascular outcomes

A total of 224 cardiovascular outcomes were observed during the follow-up. Detailed information on cardiovascular outcomes is presented in S1 Table. Univariate and multivariate Cox regression analysis demonstrated that TAC trough levels, TAC dose, age, sex, BMI, primary kidney disease, smoking, serum total cholesterol, HDL, and systolic BP were not significantly associated with cardiovascular composite endpoints (Table 4). Multivariate Cox regression analysis showed that dialysis duration was an independent predictor of the development of cardiovascular outcomes (HR = 1.01, 95% CI 1.00–1.01, P < 0.001).

**Table 4:** Univariate and multivariate Cox regression analysis for cardiovascular composite endpoints.

	Univariate		Multivariate	
	HR (95% CI)	P value	HR (95% CI)	P value
TAC > 5.9 vs. TAC ≤ 5.9, ng/mL	0.80 (0.62–1.04)	0.102	0.76 (0.58–1.01)	0.055
TAC dose, mg	0.99 (0.93–1.05)	0.642	0.99 (0.93–1.05)	0.658
Age, years	1.01 (1.00–1.02)	0.171	1.01 (0.99–1.02)	0.270
Men vs. women	1.46 (1.10–1.94)	0.010	1.31 (0.90–1.90)	0.161
BMI, kg/m^2^	1.02 (0.98–1.05)	0.420	0.98 (0.94–1.03)	0.475
Primary kidney disease				
Diabetes	1.15 (0.83–1.59)	0.397	1.08 (0.76–1.53)	0.656
Non-diabetes	Reference		Reference	
Unknown	1.35 (0.94–1.93)	0.107	1.61 (0.80–1.69)	0.437
Dialysis duration, months	1.01 (1.00–1.01)	< 0.001	1.01 (1.00–1.01)	< 0.001
Smoking at KT				
Never	Reference		Reference	
Current or former	1.28 (0.98–1.66)	0.071	1.07 (0.77–1.48)	0.694
Missing	2.34 (0.58–9.48)	0.235	1.79 (0.24–13.61)	0.573
Total cholesterol at 1 year, mg/dL	1.00 (1.00–1.01)	0.314	1.00 (1.00–1.01)	0.103
HDL at 1 year, mg/dL	1.00 (0.99–1.00)	0.360	1.00 (0.99–1.01)	0.530
SBP at 1 year, mmHg	1.01 (1.00–1.02)	0.086	1.01 (1.00–1.02)	0.119

Abbreviations: BMI, body mass index; CI, confidence interval; HDL, high-density lipoprotein; HR, hazards ratio; KT, kidney transplantation; SBP, systolic blood pressure; TAC, tacrolimus.

No significant difference in the development of cardiovascular outcomes between the HL-TAC and LL-TAC groups was observed in the Kaplan-Meier plot (Fig 2B).

### TAC trough levels and opportunistic infections

During the follow-up, 5 BK virus infections and 60 CMV infections occurred after 1 year post-transplant. Univariate and multivariate Cox regression analysis demonstrated that TAC trough levels, age, sex, BMI, number of HLA mismatches, re-transplantation, and desensitization were not significantly associated with opportunistic infections (Table 5). TAC dose was an independent risk factor for the development of opportunistic infections.

**Table 5:** Univariate and multivariate Cox regression analysis for opportunistic infections.

	Univariate		Multivariate	
	HR (95% CI)	P value	HR (95% CI)	P value
TAC ≤ 5.9 vs. TAC > 5.9, ng/mL	0.89 (0.54–1.44)	0.622	1.00 (0.60–1.64)	0.986
TAC dose, mg	1.11 (1.03–1.20)	0.009	1.11 (1.02–1.21)	0.013
Age, years	0.99 (0.97–1.02)	0.568	0.99 (0.97–1.02)	0.568
Men vs. women	1.09 (0.65–1.81)	0.746	1.19 (0.69–2.04)	0.529
BMI, kg/m^2^	0.99 (0.92–1.06)	0.683	0.98 (0.90–1.06)	0.534
Number of HLA mismatches				
Total	1.17 (0.99–1.37)	0.059	1.17 (0.89–1.54)	0.252
DR	1.30 (0.90–1.86)	0.159	0.94 (0.50–1.75)	0.841
Deceased vs. living donor	0.75 (0.38–1.46)	0.391	0.92 (0.45–1.88)	0.817
Re-transplantation	0.21 (0.03–1.51)	0.121	0.18 (0.03–1.33)	0.094
Desensitization	1.50 (0.89–2.53)	0.126	1.55 (0.89–2.71)	0.123

Abbreviations: BMI, body mass index; CI, confidence interval; HLA, human leukocyte antigen; HR, hazards ratio; TAC, tacrolimus.

### TAC trough levels, de novo DSAs, and renal allograft function

**Fig 3:**
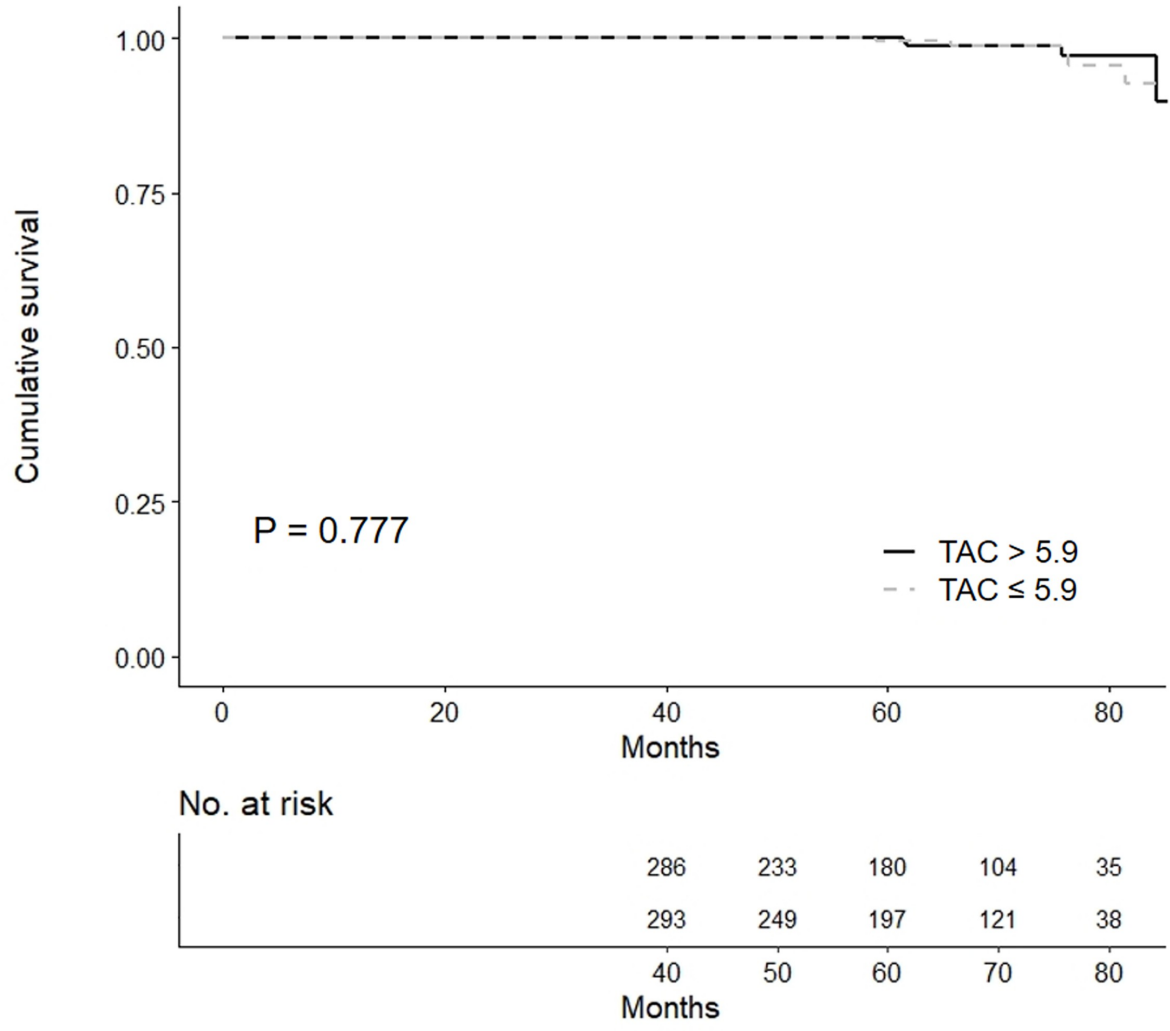
Development of de novo DSAs after 1 year post-transplant according to TAC trough levels. No significant difference in de novo DSA development between the HL-TAC and LL-TAC groups was observed in the Kaplan-Meier plot. Abbreviations: DSA, donor-specific anti-HLA antibody; LL, low-level; HL, high-level; TAC, tacrolimus.

No significant difference in the development of de novo DSAs between the HL-TAC and LL-TAC groups was observed in the Kaplan-Meier plot (Fig 3).

**Fig 4:**
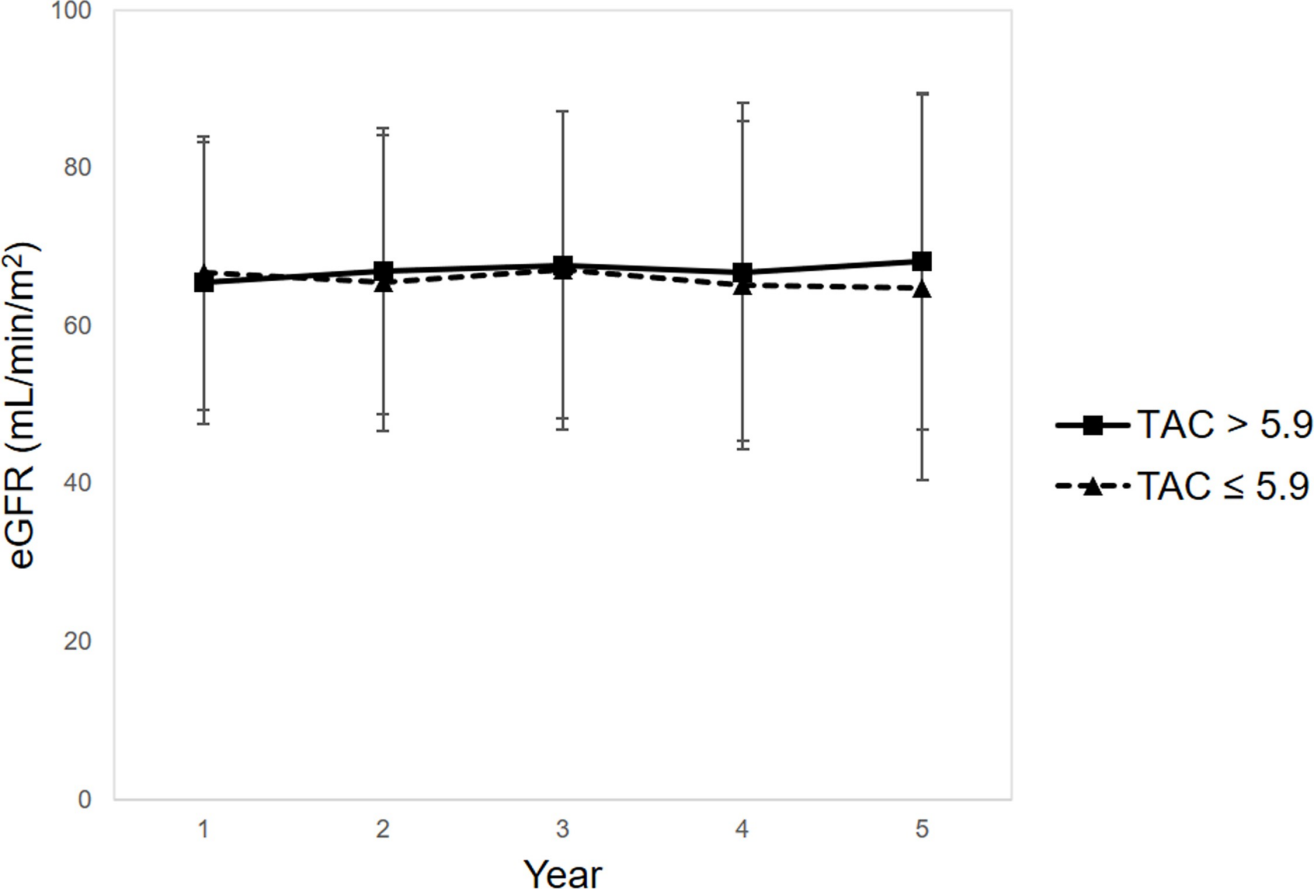
eGFRs at 2, 3, 4, and 5 years post-transplant. There were no significant differences in eGFRs at 2 years (67.0 ± 18.1 vs. 65.5 ± 18.8 mL/min/1.73 m^2^, P = 0.648), 3 years (67.8 ± 19.4 vs. 67.1 ± 20.2 mL/min/1.73 m^2^, P = 0.662), 4 years (66.9 ± 21.4 vs. 65.2 ± 20.8 mL/min/1.73 m^2^, P = 0.417), and 5 years (68.3 ± 21.3 vs. 64.9 ± 24.4 mL/min/1.73 m^2^, P = 0.273) between the HL-TAC and LL-TAC groups. Abbreviations: eGFR, estimated glomerular filtration rate; LL, low-level; HL, high-level; TAC, tacrolimus.

There were no significant differences in eGFRs at 2 years (67.0 ± 18.1 vs. 65.5 ± 18.8 mL/min/1.73 m^2^, P = 0.648), 3 years (67.8 ± 19.4 vs. 67.1 ± 20.2 mL/min/1.73 m^2^, P = 0.662), 4 years (66.9 ± 21.4 vs. 65.2 ± 20.8 mL/min/1.73 m^2^, P = 0.417), and 5 years (68.3 ± 21.3 vs. 64.9 ± 24.4 mL/min/1.73 m^2^, P = 0.273) between the HL-TAC and LL-TAC groups (Fig 4).

## Discussion

In this multicenter cohort study, TAC trough levels after 1 year post-transplant remained at a similar level until the fifth year after kidney transplantation. No significant association was found between 1-year post-transplant TAC trough levels and long-term renal and cardiovascular outcomes and opportunistic infections in stable Korean KTRs who did not experience renal and cardiovascular outcomes within 1 year post-transplant. Furthermore, TAC trough levels of > 5.9 ng/mL compared with ≤ 5, 5.0–5.9, and ≤ 5.9 ng/mL did not show any benefits in terms of outcomes as well as the development of de novo DSAs and renal allograft function. Subgroup analysis including deceased donor or deceased donor with donor age ≥ 60 also showed consistent results.

There are limited data on stable KTRs, especially in the Asian population, who have not experienced any outcomes including rejection in the first year following kidney transplantation. In this study, TAC trough levels higher than approximately 6 ng/mL did not exhibit any beneficial effect on reducing renal outcomes including the development of de novo DSAs in stable KTRs. Although an existing large-scale study reported TAC trough levels > 5 ng/mL after 1 year post-transplant as optimal for preventing the development of de novo DSAs, the study differed considerably from this study in that it included all patients who experienced BPAR within a year after kidney transplantation [19]. As BPAR itself can affect transplant outcomes and the target range of TAC trough levels, this study excluded those with clinical events within 1 year. Furthermore, TAC trough levels < 5 ng/mL in patients with low HLA alloimmune response did not increase the risk of de novo DSA development, indicating that not all KTRs may have a standard TAC trough level target of above 5 ng/mL [19]. Therefore, the TAC trough level target should be adjusted based on previous BPAR history as well as the individual’s alloimmune risk.

CNIs have been reported to cause dyslipidemia and vascular calcification dose-dependently, and these conditions may increase cardiovascular outcomes [6]. However, the reduction of cardiovascular outcomes by optimal TAC trough levels has not been investigated. Contrary to our expectations, high TAC trough levels did not increase the risk of cardiovascular outcomes in this study. Instead, pretransplant dialysis duration was an independent risk factor for cardiovascular outcomes even after adjusting for Framingham risk factors. The result of the present study is consistent with that of a previous study, which reported pretransplant dialysis duration as an independent predictor of patient death resulting from cardiovascular diseases [24]. The pathogenesis may be attributed to the rapid progression of cardiovascular changes including vascular calcification and left ventricular hypertrophy [25], increased inflammation [26], and changes in concentrations of advanced glycosylation end products [27].

This study has some limitations. First, this study was not a randomized controlled study but an observational study. The results of our study are not completely unaffected by the physician’s strategies and clinical circumstances of KTRs. Second, as the TAC trough level involved a single drug concentration, there may be a lack of representation. However, KTRs with HL-TAC at 1 year post-transplant also had significantly higher TAC trough levels until 5 years post-transplant. The sustained higher or lower TAC trough levels for the following 5 years may be explained by individual genetic predispositions, as reported in previous studies about the association between cytochromeP450(CYP)3A5 gene polymorphisms, the dose requirement of TAC, and TAC trough levels [28–30]. CYP3A5 is the major enzyme responsible for the metabolisms TAC. The CYP3A5 genetic polymorphisms can affect the pharmacokinetics of TAC and contribute to the interindividual variability of TAC trough levels. Previous studies reported that CYP3A5 genotype is associated with dose-adjusted level of TAC and complications among Korean KTRs [30, 31]. Although no information on CYP3A5 gene polymorphism has been obtained from the current study, further studies regarding the genotype‐guided TAC dosage control will ultimately be necessary to achieve individual optimized TAC trough levels and achieve the optimal transplant outcomes. Third, there are no data on the presence of proteinuria over time, which may have yielded more useful information for the evaluation of recurrent or newly occurring glomerulonephritis after kidney transplantation which are a frequent cause of allograft loss at 10 years [32]. Despite these limitations, this study could provide Asian clinicians with some guidance about TAC trough levels after 1 year post-transplant because it included an Asian population, in contrast to prior studies that included Caucasian, African American, and Hispanic populations. Furthermore, the results of this study are clinically significant, demonstrating that stable KTRs who have never experienced renal or cardiovascular outcomes make up the largest number of patients in clinical practice. In addition, the mean follow-up duration was considerably long.

In conclusion, TAC trough levels after 1 year post-transplant were not directly associated with long-term outcomes in stable Korean KTRs who did not experience renal or cardiovascular outcomes. Therefore, in Asian KTRs who have not experienced any outcomes in the first year, TAC trough levels higher than approximately 6 ng/mL might not be required after a year of kidney transplantation.

## Supporting information

S1 Table Number of renal and cardiovascular outcomes after 1 year post-transplant. (DOCX)

S2 Table Multivariate Cox regression analysis for BPRA or IFTA. (DOCX)

S1 File. Raw data (XLSX)
