# Supplementary material for: Tacrolimus trough levels higher than 6 ng/mL might not be required after a year in stable kidney transplant recipients
Source: PLoS One. 2020 Jul 2;15(7):e0235418. doi: 10.1371/journal.pone.0235418 (PMC7332007; doi:10.1371/journal.pone.0235418)
Supplement: S1 Table — (DOCX) [file pone.0235418.s001.docx]

S1 Table. Number of renal and cardiovascular outcomes after 1 year post-transplant.

| Renal outcome, n | | | |
| --- | --- | --- | --- |
| BPAR | IFTA | DCGL | Total number of renal outcomes |
| 102 | 31 | 15 | 121 |
| Cardiovascular outcome, n | | | |
| Cardiovascular events | De novo LVH | De novo cardiomegaly | Total number of cardiovascular outcomes |
| 166 | 96 | 9 | 224 |

Abbreviations: BPAR, biopsy-proven acute rejection; DCGL, death-censored graft loss; IFTA, interstitial fibrosis and tubular atrophy; LVH, left ventricular hypertrophy
