# Supplementary material for: Tacrolimus trough levels higher than 6 ng/mL might not be required after a year in stable kidney transplant recipients
Source: PLoS One. 2020 Jul 2;15(7):e0235418. doi: 10.1371/journal.pone.0235418 (PMC7332007; doi:10.1371/journal.pone.0235418)
Supplement: S2 Table — (DOCX) [file pone.0235418.s002.docx]

S2 Table. Multivariate Cox regression analysis for BPRA or IFTA.

|  | BPRA |  | IFTA |  |
| --- | --- | --- | --- | --- |
|  | AHR (95% CI) | P value | AHR (95% CI) | P value |
| TAC ≤ 5.9 vs. TAC > 5.9, ng/mL | 1.12 (0.72–1.76) | 0.613 | 0.46 (0.11–2.04) | 0.308 |
| TAC dose, mg | 1.06 (0.97–1.15) | 0.232 | 1.00 (0.78–1.29) | 0.991 |
| Age, years | 1.00 (0.98–1.02) | 0.916 | 0.93 (0.87–0.99) | 0.036 |
| Men vs. women | 1.15 (0.71–1.87) | 0.565 | 5.06 (0.60–42.89) | 0.137 |
| BMI, kg/m^2^ | 1.05 (0.98–1.12) | 0.182 | 0.96 (0.77–1.20) | 0.712 |
| Number of HLA mismatches |  |  |  |  |
| Total | 1.20 (0.94–1.53) | 0.144 | 0.68 (0.32–1.46) | 0.323 |
| DR | 0.77 (0.44–1.35) | 0.362 | 1.33 (0.20–8.92) | 0.772 |
| Deceased vs. living donor | 0.90 (0.45–1.66) | 0.732 | 2.75 (0.44–17.02) | 0.278 |
| Re-transplantation | 0.81 (0.29–2.24) | 0.681 | 1.32 (0.15–11.92) | 0.806 |
| Desensitization | 1.24 (0.73–2.10) | 0.423 | 2.41 (0.50–11.74) | 0.275 |

Abbreviations: AHR, adjusted hazards ratio; BMI, body mass index; BPAR, biopsy-proven acute rejection; CI, confidence interval; HLA, human leukocyte antigen; IFTA, interstitial fibrosis and tubular atrophy; TAC, tacrolimus
